# 

**Published:** 1873-05

**Authors:** 


MAY, 1873.

### THE AMERICAN JOURNAL OF DENTAL SCIENCE,

EDITED BY

F. J. S. GORGAS, M. D., D. D. S.

VOL. 7.—THIRD SERIES.—NO. 1.

BALTIMORE:
SNOWDEN & COWMAN.
LONDON:
TRUBNER & CO., 60 PATERNOSTER ROW.
1873.

$2.50 PER ANNUM.

PAYABLE IN ADVANCE.

